# Fatal Epstein-Barr Virus Reactivation in an Acquired Aplastic Anemia Patient Treated with Rabbit Antithymocyte Globulin and Cyclosporine A

**DOI:** 10.1155/2015/926874

**Published:** 2015-09-03

**Authors:** Tohru Takahashi, Yumiko Maruyama, Mayuko Saitoh, Hideto Itoh, Mitsuru Yoshimoto, Masayuki Tsujisaki

**Affiliations:** ^1^Department of Hematology, Tenshi Hospital, Kita-12, Higashi-3-1-1, Higashi-ku, Sapporo 065-0012, Japan; ^2^Department of Gastroenterology, Tenshi Hospital, Kita-12, Higashi-3-1-1, Higashi-ku, Sapporo 065-0012, Japan

## Abstract

Epstein-Barr virus (EBV) associated lymphoproliferative disorder (LPD) after immunosuppressive therapy for aplastic anemia (AA) is extremely rare in a nontransplant setting and has not been well described. This report describes a severe AA patient in whom fatal EBV-LPD developed after being treated with rabbit antithymocyte globulins (ATG) and cyclosporine A (CsA). An 81-year-old man was diagnosed as having severe AA. He was started on CsA followed by administration of ATG for five consecutive days. One month after the start of ATG, persistent fever which was not responsive to antibiotics or antifungal agents developed and atypical lymphocytes emerged in peripheral blood. Repeated blood cultures were negative. An extremely high level of EBV virus in his peripheral blood plasma was detected by means of a quantitative real-time PCR assay. Even after the cessation of CsA, the fever persisted and the peripheral atypical lymphocytes proliferated rapidly. The patient suffered from respiratory failure, liver dysfunction, and metabolic acidosis. Rituximab was administered without success and he died.

## 1. Introduction

Acquired aplastic anemia (AA) is a rare disease characterized by pancytopenia associated with hypocellular bone marrow. Without treatment, it is often fatal in its severe form. Most of AA is considered an immune-mediated disease because the patients show hematological response after transient T-cell reduction by antithymocyte globulins (ATG) [1]. Hematopoietic stem cell transplantation (HSCT) can cure severe AA and is a standard therapy for younger patients with a suitable donor. For older or HSCT ineligible severe AA patients, immunosuppressive therapy (IST) using ATG and cyclosporine A (CsA) is also a standard treatment.

Epstein-Barr virus (EBV) related diseases have been increasingly recognized in immune deficient hosts. Patients who underwent allogeneic HSCT are prone to EBV reactivation and development of EBV-associated lymphoproliferative disorder (LPD) [2]. In particular, T-cell depleted transplantation and use of ATG were predictive factors for developing EBV-LPD in the patients. Though EBV-LPD is rare complication, it sometimes becomes fatal. It is reported that reactivation of EBV was also observed in the majority of severe AA patients treated with ATG [3]. However, EBV-LPD after IST in severe AA is extremely rare in a nontransplant setting, and it has not been well described. Here we report a case of fatal EBV-LPD developed in an 81-year-old patient with severe AA, after being treated with rabbit ATG and CsA.

## 2. Case Presentation

An 81-year-old man was admitted to our hospital in September 2014 because of pancytopenia. He complained of no subjective symptoms and had been doing fine. Pancytopenia was pointed out on a routine periodic check-up at a hospital where he underwent coronary artery stenting two years earlier. He also had been treated with type 2 diabetes mellitus and hypertension for several years. He underwent a surgery for lumbar spinal canal stenosis ten years earlier and a transurethral prostate resection for the prostate cancer eight years earlier. On physical examination, his conjunctiva was anemic. His superficial lymph nodes were not palpated. The liver and spleen were not palpated. His lung was clear and no abnormal cardiac sounds were heard on auscultation. There were several small purpura in his torso. He was afebrile.

**Figure 1:**
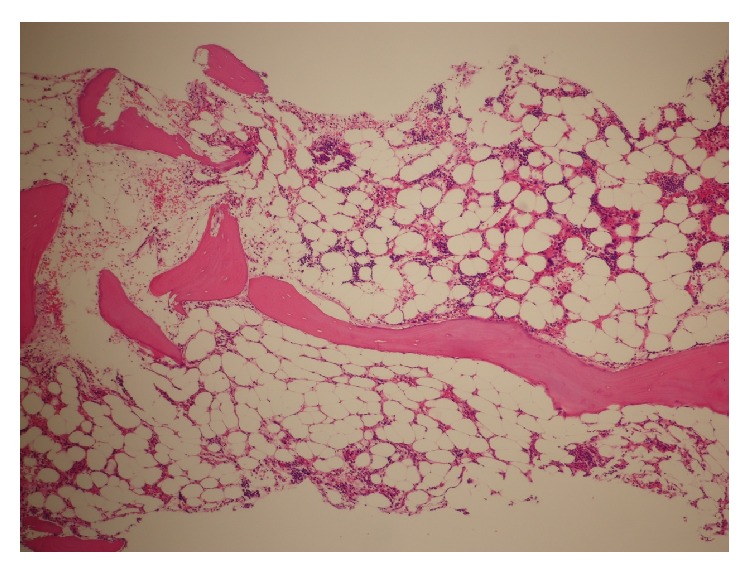
Bone marrow biopsy. The marrow was hypoplastic with no myelofibrosis.

The white cell count was 1, 730/*μ*L with 44.5% neutrophils, 6.0% monocytes, 0.5% eosinocytes, 0.5% basophils, and 48.5% lymphocytes. There were no atypical lymphocytes. The hemoglobin concentration was 5.7 g/dL, and the platelet count was 4,000/*μ*L. Absolute reticulocyte count was 18,600/*μ*L. Results of serum biochemistry tests were normal. A bone marrow biopsy showed that the marrow was hypoplastic with no myelofibrosis or no lymphocytosis (Figure 1). Myelodysplasia or atypical lymphocytes were not observed by a bone marrow aspiration study. A chromosomal analysis of the marrow showed a normal karyotype. A whole-body CT scan showed no hepatosplenomegaly or internal lymphadenopathy. Thus, a diagnosis of severe AA was made.

**Figure 2:**
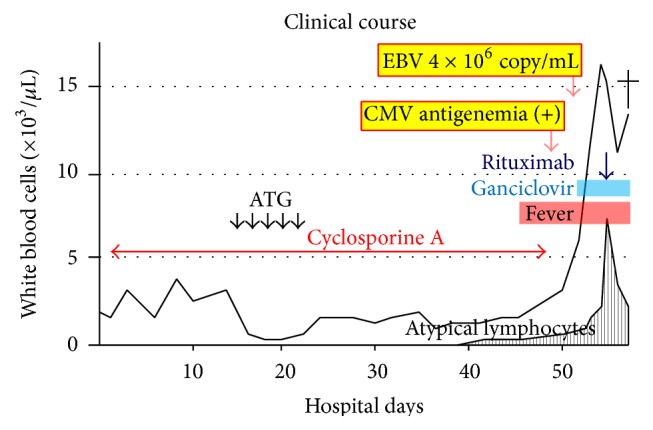
Clinical course.

**Figure 3:**
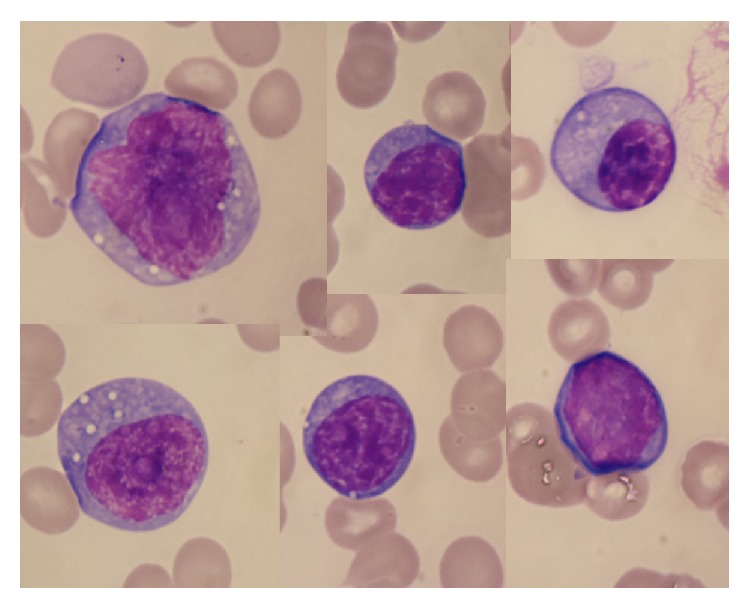
Atypical lymphocytes in the peripheral blood.

He was started on CsA since the 2nd hospital day. Frequent transfusions of red blood cells and platelets and daily administration of G-CSF became necessary. Rabbit ATG were started on the 15th hospital day at a dose of 3.3 mg/kg/day for 5 consecutive days, and severe leukocytopenia followed (Figure 2). On the 46th hospital day, the patient developed a persistent high-grade fever. Repeated blood cultures were negative, and antibiotics or antifungal drugs were ineffective. The fever persisted even after his white blood cells were recovered (2,730/*μ*L with 75.5% neutrophils and 4.5% atypical lymphocytes on the 49th hospital day). Atypical lymphocytes in the peripheral blood proliferated gradually (Figure 2). CsA was discontinued since a reactivation of EBV was suspected. Superficial lymphadenopathy or hepatosplenomegaly was not detected by a physical examination. The patient was started on ganciclovir at the 51st hospital day because reactivation of CMV was also shown by means of a CMV antigenemia assay on the 49th hospital day. A quantitative real-time PCR assay of the patient's plasma showed 4 × 10^6^ EBV-DNA copies/mL (on the 51st hospitals day). The atypical lymphocytes were mainly lymphoplasmacytoid cells, and a few of them were large transformed cells with inconspicuous nucleoli (Figure 3). A flow cytometry of circulating lymphocytes revealed 70.1% CD19+ cells, 35.6% CD20+ cells, 29.4% CD3+ cells, 6.8% CD4+ cells, 27.1% CD8+ cells, 23.9% kappa chain+ cells, 10.1% lambda chain+ cells, 14.3% CD15+ cells, and 22.5% CD30+ cells. Therefore the majority of atypical lymphocytes were confirmed to be of B-cell origin. Though we did not perform in situ hybridization to confirm integration of EBV-DNA in the cells, we considered that a diagnosis of EBV-LPD was appropriate because of the extremely high viral load of EBV in the patient's plasma. Organ biopsies or imaging studies with a CT scan were not available at that time due to the poor patient condition. Respiratory failure developed on the 51st hospital day and severe metabolic acidosis followed on the 54th hospital day. On the 56th hospital day, WBC increased to 15,370/*μ*L with 48.0% atypical lymphocytes and the serum LDH increased to 1,367 IU/L (reference range, 115–245). Soluble IL-2 receptor level was very high as 5,473 U/mL (135–483), and serum ferritin level was also extremely high as 23,892 ng/mL (21–282). Rituximab was administered, but general condition of the patient was not improved. He died on the 58th hospital day.

## 3. Discussion

Our patient presented with pancytopenia and his bone marrow was hypoplastic. The chromosomal analysis was normal. There were no atypical lymphocytes in the peripheral blood or bone marrow at the presentation. He did not have fever, lymphadenopathy, or hepatosplenomegaly. Therefore his pancytopenia was not caused by a hemophagocytic lymphohistiocytosis, which might have developed later in the disease course as he had high fever and hyperferritinemia. Reactivation of EBV as well as CMV occurred after the IST with CsA and ATG. Rapid proliferation of atypical B-cells was observed and he died. EBV-LPD was considered to have developed because of severe immunosuppression caused by the CsA and the rabbit ATG. Clonality of the cells was not shown by the flow cytometry. The atypical lymphocytes might have been oligoclonal. EBV-driven B-lymphocytosis is not necessary to be monoclonal.

Posttransplant LPD is lymphoid or plasmacytic proliferations that develop as a consequence of immunosuppression in patients who underwent solid organ transplantation or HSCT. They comprise a spectrum ranging from usually EBV-driven infectious mononucleosis-type polyclonal proliferations to EBV positive or negative proliferations indistinguishable from lymphomas that occur in immunocompetent hosts [11]. EBV-LPD in patients undergoing allogeneic HSCT is increasingly recognized because stronger IST including ATG or alemtuzumab is being used in the conditioning regimen before HSCT to reduce the risk of acute or chronic graft versus host diseases. However, development of EBV-LPD after IST for severe AA in nontransplant setting is extremely rare. Scheinberg et al. reported that none of the 78 patients in their study with severe AA developed EBV-LPD [3].

Seven cases of EBV-LPD after ATG therapy for AA have been reported to date [4–10] (Table 1). The signs and symptoms were resolved after cessation of IST in 2 cases [8, 10]. Rituximab was used in other three cases after cessation of IST, and cyclophosphamide was added in one of the three cases [5–7]. The diseases were well controlled and did not recur in those cases. There were two cases of EBV-LPD after IST for severe AA with fatal outcome [4, 9]. Ohata et al. reported a case of EBV-LPD that occurred 3 weeks after the use of rabbit ATG administered for severe hepatitis-associated AA [9]. The patient died of fulminant leukemic lymphoma associated with respiratory failure and metabolic acidosis. Clinical course of this case seems to have some similarity with our case.

Rabbit ATG was used in 6 cases including our case, and horse ATG was administered in three cases. Both horse and rabbit ATG were used nonconcurrently in two cases. Case 1 was refractory of previous course of IST including ATG and CsA, and horse ATG was used in the second course of IST with CsA, methylprednisolone, and IL-3. Case 4 received horse ATG alone, but he had a history of Burkitt-like lymphoma and underwent autologous HSCT. It was reported that lymphopenia was more protracted in patients treated with rabbit ATG than in patients who received horse ATG [12], and it has been estimated that rabbit ATG shows more immunosuppressive efficacy than horse ATG [13]. Therefore, the risk of EBV-LPD might be higher in the AA patients treated with rabbit ATG than with horse ATG. Moreover, other factors such as the second course of ATG or previous history of malignancy should be also considered as a risk factor. The rabbit ATG was used in our case at a dose of 3.3 mg/kg/day (recommended dose by manufacturer is 2.5–3.75 mg/kg/day) for 5 consecutive days. Smaller dose of the ATG might have circumvented the reactivation of EBV in our case, considering his age of 81.

Wagner et al. recommend quantitative measurement of EBV-load to enable prompt treatment of EBV-LPD with anti-CD20 monoclonal antibody or EBV-specific CTL for patients who underwent T-cell depleted HSCT [14]. On the contrary, in nontransplant setting, Scheinberg et al. conclude that there is no need to routinely monitor viral reactivation in patients with severe AA following antibody-based IST regimens [3]. The fact that increasing cases of EBV-LPD after IST for severe AA has been reported (Table 1) might argue against their conclusion. Wondergem et al. suggest the usefulness of monitoring EBV reactivation in patients being treated with rabbit ATG as a second course of IST [6]. Ohata et al. suggested that once-a-week screening of EBV would have been useless for their patient because of rapid proliferation of EBV and the prompt examination of EBV copy number in response to clinical signs such as pyrexia may be more practical for appropriately starting rituximab [9]. We completely agree with their suggestion. Though immediate intervention is crucial because of the rapid clinical course of EBV-LPD, weekly monitoring of EBV-load is not practical. Our patient might have been rescued if rituximab was administered preemptively in the early phase of EBV-LPD. EBV-viral load should be examined promptly after the onset of fever or emergence of atypical lymphocytes.

Herein, we reported a severe AA patient in whom fatal EBV-LPD developed after being treated with rabbit ATG and CsA. Practical guideline to prevent development of fatal EBV-LPD after IST of severe AA is desired.

## Figures and Tables

**Table 1:** Reported cases of EBV-LPD after ATG therapy for aplastic anemia.

Case number	Age/sex	Source	Other IST	Histopathology	Therapy	Outcome	References
1	36/M	NA→H	CsA	EBV-LPD	None	Dead	[4]
2	38/M	R→H	CsA	NA (IM)	Rit.	Resolved	[5]
3	42/F	H→R	CsA	DLBCL	Rit., CPM	Resolved	[6]
4	55/M	H	None	Plasma cell hyperplasia	Rit.	Resolved^*∗*^	[7]
5	55/M	R	CsA	EBV-LPD (colon)	None	Resolved	[8]
6	54/M	R	CsA	DLBCL	None	Dead	[9]
7	46/F	R	CsA	Plasmacytic-LPD	None	Resolved	[10]
Present case	81/M	R	CsA	NA	Rit.	Dead	

H: horse; R: rabbit; NA: not available; IM: infectious mononucleosis; DLBCL: diffuse large B-cell lymphoma; Rit.: rituximab; CPM: cyclophosphamide.

^*∗*^The patient developed acute myeloid leukemia later and died.
